# New therapeutic strategy of hinokitiol in haemorrhagic shock‐induced liver injury

**DOI:** 10.1111/jcmm.14070

**Published:** 2018-12-08

**Authors:** Wan‐Jung Lu, Kuan‐Hung Lin, Mei‐Fang Tseng, Kuo‐Ching Yuan, Hung‐Chang Huang, Joen‐Rong Sheu, Ray‐Jade Chen

**Affiliations:** ^1^ Department of Medical Research Taipei Medical University Hospital Taipei Taiwan; ^2^ Department of Pharmacology School of Medicine College of Medicine Taipei Medical University Taipei Taiwan; ^3^ Graduate Institute of Metabolism and Obesity Sciences College of Public Health and Nutrition Taipei Medical University Taipei Taiwan; ^4^ Central Laboratory Shin‐Kong Wu Ho‐Su Memorial Hospital Taipei Taiwan; ^5^ Institute of Biomedical Sciences Mackay Medical College New Taipei City Taiwan; ^6^ Department of Emergency and Critical Care Medicine and Division of Acute Care Surgery and Trauma Department of Surgery Taipei Medical University Hospital Taipei Taiwan; ^7^ Division of General Surgery Department of Surgery Taipei Medical University Hospital Taipei Taiwan; ^8^ Department of Surgery School of Medicine College of Medicine Taipei Medical University Taipei Taiwan; ^9^ Graduate Institute of Medical Sciences Taipei Medical University Taipei Taiwan

**Keywords:** Hemorrhagic shock, hinokitiol, liver, resuscitation, trauma

## Abstract

Haemorrhagic shock and resuscitation (HS/R) may cause global ischaemia‐reperfusion injury, which can result in systemic inflammation, multiorgan failure (particularly liver failure) and high mortality. Hinokitiol, a bioactive tropolone‐related compound, exhibits antiplatelet and anti‐inflammatory activities. Targeting inflammatory responses is a potential strategy for ameliorating hepatic injury during HS/R. Whether hinokitiol prevents hepatic injury during HS/R remains unclear. In the present study, we determined the role of hinokitiol following HS/R. The in vivo assays revealed that hinokitiol markedly attenuated HS/R‐induced hepatic injury. Hinokitiol could inhibited NF‐κB activation and IL‐6 and TNF‐α upregulation in liver tissues. Moreover, hinokitiol reduced caspase‐3 activation, upregulated Bax and downregulated Bcl‐2. These findings suggest that hinokitiol can ameliorate liver injury following HS/R, partly through suppression of inflammation and apoptosis. Furthermore, the in vitro data revealed that hinokitiol significantly reversed hypoxia/reoxygenation (H/R)‐induced cell death and apoptosis in the primary hepatocytes. Hinokitiol prevented H/R‐induced caspase‐3 activation, PPAR cleavage, Bax overexpression and Bcl‐2 downregulation. Moreover, hinokitiol attenuated H/R‐stimulated NF‐κB activation and reduced the levels of IL‐6 and TNF‐α mRNAs, suggesting that hinokitiol can protect hepatocytes from H/R injury. Collectively, our data suggest that hinokitiol attenuates liver injury following HS/R, partly through the inhibition of NF‐κB activation.

## 1 INTRODUCTION

According to a study by the World Health Organization, trauma accounts for 10% of deaths and 16% of disabilities worldwide.^1^ Haemorrhagic shock (HS) is the most common preventable cause of death after trauma.^1^, ^2^ If uncontrolled bleeding occurs, fluid resuscitation after HS (HS/R) is required to prevent tissue hypoxia, inflammation and organ dysfunction. However, fluid resuscitation causes dilution of clotting factors and increased leucocyte‐endothelial interaction, thereby exacerbating coagulopathy and inflammation.^3^ Moreover, reperfusion also cause additional injury involving the generation of reactive oxygen species. Thus, restrictive fluid resuscitation has been adopted as the standard care in many trauma systems.^3^

Severe tissue injury after trauma can cause systemic inflammatory response syndrome (SIRS) resulting from the release of damage‐associated molecular patterns (DAMPs), which can trigger TLR4 and nuclear factor (NF)‐κB activation and subsequently increase the expression of pro‐inflammatory cytokines, such as interleukin (IL)‐6 and tumour necrosis factor (TNF)‐α, eventually leading to multi‐organ failure.^4^, ^5^ Among the organs, the liver is currently considered to be the most frequently affected by HS.^6^ Liver dysfunction following HS/R results from a combination of ischaemia and reperfusion injury that has been reported to cause massive production of pro‐inflammatory mediators and subsequent accumulation of neutrophils in the liver, which is directly correlated with mortality.^6^ Thus, targeting inflammatory responses might be a potential strategy to ameliorate hepatic injury during HS/R.

**Figure 1:**
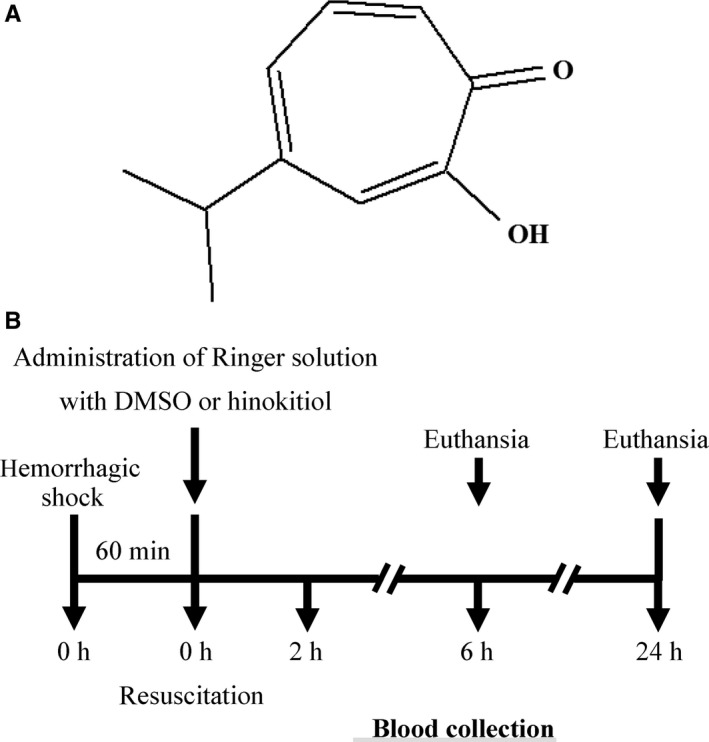
Timeline of haemorrhagic shock (HS)/resuscitation (R). A, Chemical structure of hinokitiol. B, Hinokitiol was administered 1 h after haemorrhagic shock (HS1). Then, blood was collected 2, 6 and 24 h after resuscitation (R2, R6 and R24, respectively). The mice were sacrificed after HS1/R6 and HS1/R24

Hinokitiol (4‐isopropyl‐tropolone; β‐thujaplicin, Figure 1A) is a bioactive tropolone‐related compound found in the wood of cupressaceous plants. Hinokitiol has various pharmacological activities, including antimicrobial, anticancer and anti‐inflammatory properties.^7^, ^8^, ^9^ Hinokitiol suppresses tumour growth by inhibiting cell proliferation and inducing cell differentiation and apoptosis in various carcinoma cell lines.^9^, ^10^, ^11^ Hinokitiol was also reported to exhibit anti‐inflammatory activities in various cells in response to lipopolysaccharide or polyriboinosinic:polyribocytidylic acid through the inhibition of NF‐κB activity.^12^ Our previous studies have also revealed that hinokitiol can attenuate platelet activation, regulate immune response and suppress tumour activity.^13^, ^14^, ^15^

In summary, because of these multiple biological activities, including antiplatelet, immune regulatory, and anti‐inflammatory activities, hinokitiol may ameliorate HS/R‐mediated hepatic injury. Moreover, our preliminary data revealed that hinokitiol reduced the levels of serum alanine amino transferase (ALT) and aspartate amino transferase (AST) during HS/R, which remain be important indicators to evaluate liver dysfunction.^16^ Thus, this result indicates that hinokitiol may prevent HS/R‐induced liver injury. Therefore, we further investigated the mechanism underlying the role of hinokitol in preventing liver injury during HS/R.

## 2 METHODS

### 2.1 Materials

Hinokitiol (99%, Figure 1A), dimethyl sulfoxide (DMSO), bovine serum albumin (BSA), collagenase type IV, and 3‐(4,5‐dimethylthiazol‐2‐yl)‐2,5‐diphenyl tetrazolium bromide (MTT) were purchased from Sigma‐Aldrich (St. Louis, MO, USA). Type I collagen was purchased from Corning (NY, USA). The β‐actin antibody, Dulbecco's modified Eagle's medium (DMEM), L‐glutamine‐penicillin‐streptomycin, fetal bovine serum, Pierce™ 1‐Step Transfer Buffer, SuperScript™ IV First‐Strand Synthesis System Kit, and Fast SYBR™ Green Master Mix were purchased from Thermo Fisher (Waltham, MA, USA). The NucleoSpin^®^ RNA kit was purchased from Macherey‐Nagel (Düren, Germany). Primary antibodies against Bax, Bcl‐2, phospho‐NF‐κB p65 (Ser^536^), IκBα, cleaved caspase‐3, and PARP were purchased from Cell Signaling (Beverly, MA, USA). The glyceraldehyde 3‐phosphate dehydrogenase (GAPDH) antibody was purchased from GeneTex (Irvine, CA, USA). Hybond‐P polyvinylidene difluoride (PVDF) membrane, the enhanced chemiluminescence (ECL) western blotting detection reagent, horseradish peroxidase (HRP)‐conjugated donkey anti‐rabbit immunoglobulin G (IgG), and the sheep anti‐mouse IgG were purchased from Amersham (Buckinghamshire, UK). CF488A Donkey anti‐mouse IgG and CF594 Donkey anti‐rabbit IgG were purchased from Biotium (Fremont, CA, USA). Hinokitiol was dissolved in 0.1% dimethyl sulfoxide (DMSO) and stored at 4°C.

### 2.2 Animals

C57BL/6 mice (20‐25 g, male, 5‐6 weeks old) were obtained from BioLasco (Taipei, Taiwan). Approval was obtained for all procedures by submitting an Affidavit of Approval of Animal Use Protocol‐Taipei Medical University (Approval No. LAC‐2016‐0081) and the procedures were conducted in accordance with the *Guide for the Care and Use of Laboratory Animals* (8th edition, 2011).

### 2.3 Isolation of primary mouse hepatocytes

The mouse hepatocyte isolation was performed using collagenase perfusion as described by Li et al.^17^ The mice were anesthetized using a mixture containing 75% air and 3% isoflurane maintained in 25% oxygen. At sterile conditions, the abdominal cavity was opened by making a *U*‐shaped incision. The intestines were moved to the left of the animal's torso in order to reveal and expose the inferior vena cava (IVC). Specifically, the portal vein was cannulated using a 22‐gauge intravenous catheter, and the liver was perfused with calcium‐free Hank's balanced salt solution (HBSS, 33 mmol/L KCl, 0.441 mmol/L KH_2_PO_4_, 4.17 mmol/L NaHCO_3_, 137.93 mmol/L NaCl, 0.338 mmol/L Na_2_HPO_4_, 4.75 mg/mL D‐glucose, 0.1 mmol/L EGTA) for about 10 minutes (5 mL/min), followed by collagenase IV (0.025%) in HBSS containing 5 mmol/L CaCl_2_ for about 8‐10 minutes (5 mL/min). All solutions were maintained at 37°C. The partially digested liver was excised, passed through a 100 μm cell strainer and resuspended in DMEM containing certified fetal bovine serum (10%). The cell suspension was filtered through the strainer, and allowed to settle for 10 minutes. The supernatant was carefully removed. The hepatocyte fraction was purified through centrifugation in ice‐DMEM at 50 g for 2 minutes at 4°C, and was then resuspended in ice‐DMEM after a second centrifugation at the same speed and temperature by using a gradient of 50% Percoll‐DMEM. The viability of the hepatocytes, which was maintained at >85% during the experiment, was determined using trypan blue exclusion.

### 2.4 Primary hepatocyte treatment

The mouse hepatocytes were cultured in DMEM supplemented with 10% fetal bovine serum. For experiments involving hypoxia, the medium was replaced with serum‐free DMEM that was equilibrated with 5% CO_2_, 90% N_2_, and 5% O_2_ and placed in a Compact Oxygen Controller (ProOx model 110; Biospherix, Lacona, NY, USA), that was flushed with an identical gas mixture. After 60 minutes of hypoxia, the cells were incubated under normoxic conditions (air/5% CO_2_) for the indicated times. The medium and cells were collected for further analysis. Cell viability was evaluated using a colorimetric assay. Cell viability was measured based on MTT assay. All experiments were performed at least three times to confirm the results.

### 2.5 Animal model and treatment

In this study, we used a mouse model of warm partial hepatic HS/R.^18^ The mice were anesthetized using a mixture containing 75% air and 3% isoflurane maintained in 25% oxygen, and cannulation of the femoral arteries and jugular vein was performed. Haemorrhagic shock (HS) was induced by controlled withdrawal of blood in order to achieve a fall in mean arterial pressure to 30 ± 2 mm Hg, and was maintained for a period of 60 minutes. After 60 minutes of HS, the mice were resuscitated using Lactated Ringers solution combined with DMSO or hinokitiol (a fixed volume of 3x each animal's shed blood volume) via a syringe pump set to dispense (over a 15 minutes) and the mice were killed at various time following reperfusion. The mice were divided into three groups (n* *=* *4 per group), namely sham‐operated, HS/R (DMSO‐treated, solvent control), and HS/R+hinokitiol‐treated (1.7 mg/kg, intravenous). Liver and serum samples were immediately collected from the mice. The sham‐operated mice underwent the same surgical procedure without vasculature occlusion. The dose of hinokitiol administered to the mice was calculated using the human dose as a reference.^19^

### 2.6 Serum sample assays and liver histology

The serum levels of ALT, AST and LDH were measured using a VetTest^®^ Chemistry Analyzer (IDEXX, Westbrook, Maine, USA). Enzyme activities were expressed as international units per liter. The mice were killed and the liver tissue of each mouse was retrieved and fixed in 10% buffered formalin. Subsequently, the liver tissues were embedded in paraffin and sectioned (1 μm). The sections were deparaffinized, rehydrated, and then stained with haematoxylin and eosin (H&E), and were used to calculate the necrotic area using a ScanScope CS (Leica Biosystems, Germany).

### 2.7 Immunofluorescent staining

In the in vitro study, the hepatocytes were washed with phosphate‐buffered saline (PBS) and then fixed using 4% paraformaldehyde in PBS for 10 minutes. After fixation, the cells were permeabilized using 0.2% Triton X‐100 for 20 minutes, and were blocked with 5% BSA in PBS for 40 minutes. The prepared samples were incubated overnight at 4°C using primary antibodies. Subsequently, the samples were washed three times with PBS and exposed to secondary antibodies for 2 hours. The samples were then counterstained with 4′,6‐diamidino‐2‐phenylindole (DAPI, 30 μmol/L) and mounted using a mounting buffer (Vector Laboratories) on a glass coverslip. The samples were observed under a Leica TCS SP5 confocal spectral microscope imaging system using an argon or krypton laser (Mannheim, Germany). Quantitative analysis of relative fluorescence was determined using ImageJ 1.48v software, and a total of 100 cells were counted during analysis.

### 2.8 Quantitative real‐time PCR

Following the manufacturer's instructions, total RNA was extracted from frozen liver tissues or primary cells using NucleoSpin^®^ RNA kit (Macherey‐Nagel, Düren, Germany) and reverse‐transcribed into cDNA using a SuperScript™ IV First‐Strand Synthesis System Kit (Thermo Fisher, Waltham, MA, USA). Quantitative real‐time PCR was performed using Fast SYBR™ Green Master Mix (Thermo Fisher, Waltham, MA, USA) to determine the expression levels of target genes, and the results were normalized against to GAPDH expression. Amplification was performed in a StepOne Real‐Time PCR systems (Applied Biosystems, Grand Island, NY, USA). The cycling conditions were: hot‐start activation at 95°C for 20 seconds, followed by 40 cycles of denaturation at 95°C for 3 seconds and annealing/extension at 60°C for 30 seconds, respectively. The following primers were used in this study: GAPDH (5′‐GAACATCATCCCTGCATCCA‐3′ and 5′‐GCCAGTGAGCTTCCCGTTCA‐3′), Bax (5′‐TGAGCGAGTGTCTCCGGCGAAT‐3′ and 5′‐GCACTTTAGTGCACAGGGCCTTG‐3′), Bcl‐2 (5′‐TGGTGGACAACATCGCCCTGTG‐3′ and 5′‐GGTCGCATGCTGGGGCCATATA‐3′), TNF‐α (5′‐CATCTTCTCAAAATTCGAGTGACAA‐3′ and 5′‐TGGGAGTAGACAAGGTACAACCC‐3′), and IL‐6 (5′‐AGTTGCCTTCTTGGGACTGA‐3′ and 5′‐TCCACGATTTCCCAGAGAAC‐3′).

### 2.9 Immunoblotting

A western blotting assay was performed using whole lysates from either liver tissue or cultured cells, as previously described.^20^ Briefly, 30 μg of extracted protein was subjected to sodium dodecyl sulfate polyacrylamide gel electrophoresis on an 8%‐12% gel and transferred to PVDF membranes. Nonspecific binding sites were blocked with TBST (10 mmol/L Tris‐base, 100 mmol/L NaCl, and 0.01% Tween 20; with 5% skim milk powder) solution for 1 hour at room temperature, and the membranes were probed with the aforementioned primary antibodies. The membranes were incubated with HRP‐conjugated anti‐mouse IgG or anti‐rabbit IgG (diluted 1:3000 in TBST) for 1 hour. Immunoreactive bands were detected using an ECL system. The ratios of the semiquantitative results were obtained by scanning the reactive bands and quantifying the optical density using a videodensitometer and the Bio‐profil Biolight software, Version V2000.01 (Vilber Lourmat, Marne‐la‐Vallée, France).

### 2.10 Immunohistochemistry

1‐μm sections were prepared from paraffin‐embedded tissues, deparaffinized in xylene, rehydrated in a graded alcohol series, and washed in deionized water. After antigen retrieval (boiling on a hotplate for 12 minutes in 10 mmol/L sodium citrate, pH 6.0), the intrinsic peroxidase activity was blocked by incubation with 3% hydrogen peroxide. Nonspecific antibody‐binding sites were blocked using 3% BSA in PBS. Sections were incubated with appropriately diluted primary anti‐bodies specific for cleaved caspase 3 for 2 hours at room temperature. After undergoing washing with PBST, the secondary antibody was applied for 1 hour at room temperature. Stained sections were detected with 3,3ʹ‐dia‐minobenzidine tetrahydrochloride (DAB), and then observed under a light microscope.

### 2.11 Data analysis

The experimental results are expressed as the means ± standard error of means and are accompanied by the number of observations (n). The values of n refer to the number of experiments performed with blood from different donors. The experimental results were evaluated using an analysis of variance (ANOVA). If ANOVA indicated significant differences between the group means, the groups were compared using the Student‐Newman‐Keuls test. The results of comparisons with *P* value of < 0.05 were considered statistically significant. All statistical analyses were performed using the SAS, Version 9.2 (SAS Inc., Cary, NC).

## 3 RESULTS

### 3.1 Hinokitiol ameliorates haemorrhagic shock/resuscitation (HS/R)‐induced hepatic injury in mice

**Figure 2:**
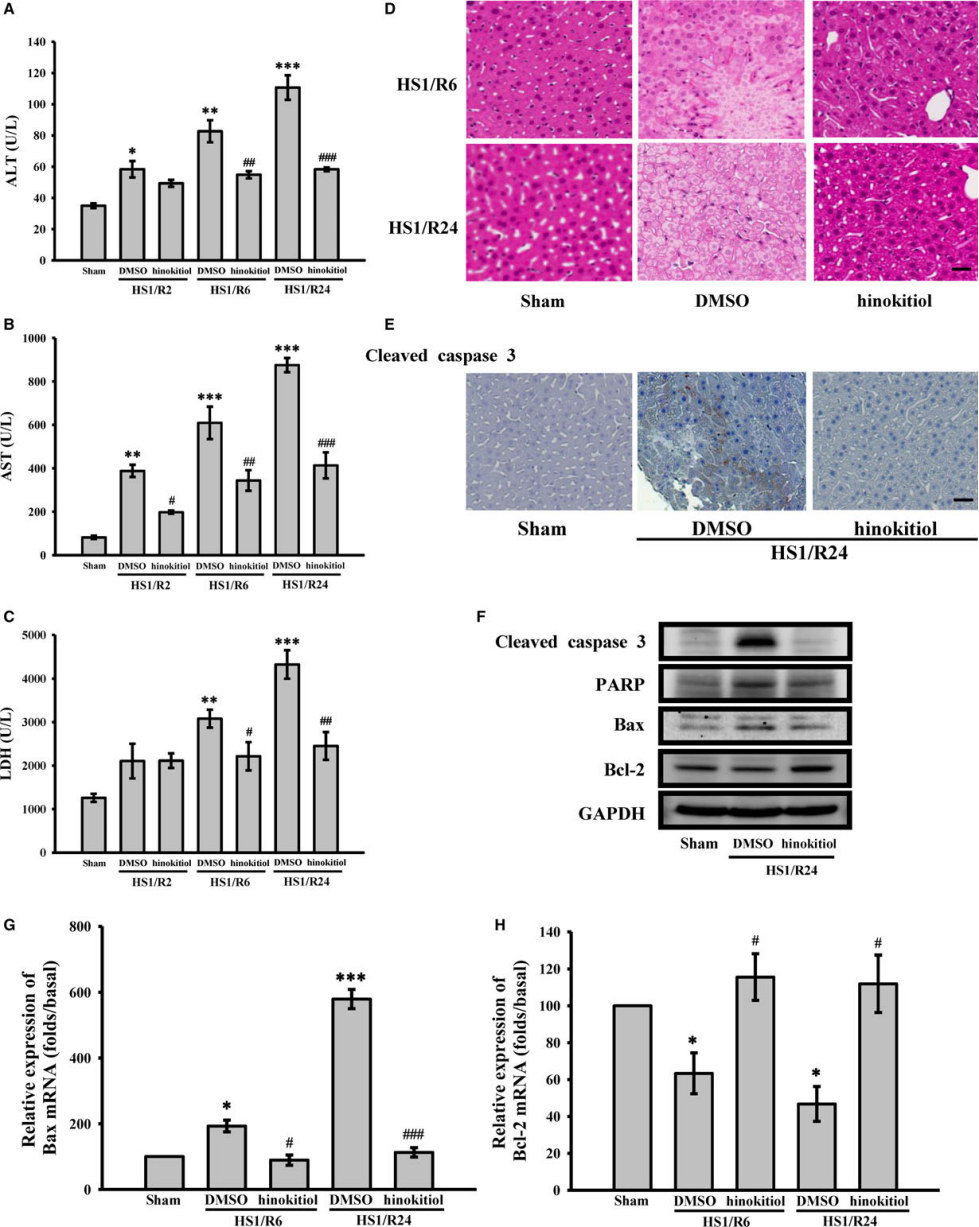
Effects of hinokitiol on HS/R‐induced hepatic injury. HS mice were treated with DMSO (solvent control) or 1.7 mg/kg hinokitiol on resuscitation. (A, B and C) Blood were collected at 2, 6 and 24 h after resuscitation (HS1/R2, HS1/R6 and HS1/R24), respectively and serum ALT, AST, and LDH were determined. (D) After being subjected to HS1/R6 and HS1/R24, the mice were sacrificed and photomicrographs of liver sections were stained with haematoxylin and eosin (H&E). (E and F) Liver section and tissue were collected, and subcellular extracts were analysed through immunohistochemical staining and immunoblotting. Specific antibodies were used to detect cleaved caspase‐3, PARP, Bax and Bcl‐2 expression. (G and H) Total RNA was extracted from liver tissue, and gene expression of Bax and Bcl‐2 was quantified by real‐time PCR. Each sample was examined in triplicate, and the amounts of the PCR products produced were normalized to glyceraldehyde 3‐phosphate dehydrogenase (GAPDH), as an internal control. Data (A, B, C, G and H) are presented as the means ± SEM (n* *=* *4). *P <* *0.05, **P <* *0.01 and ***P <* *0.001, compared with the sham‐operated group; #*P *<* *0.05, ##*P *<* *0.01 and ###*P *<* *0.001, compared with DMSO‐treated group subjected to HS/R. The profiles (D and E) are representative examples of three similar experiments. The scale bar indicates 40 μm

**Figure 3:**
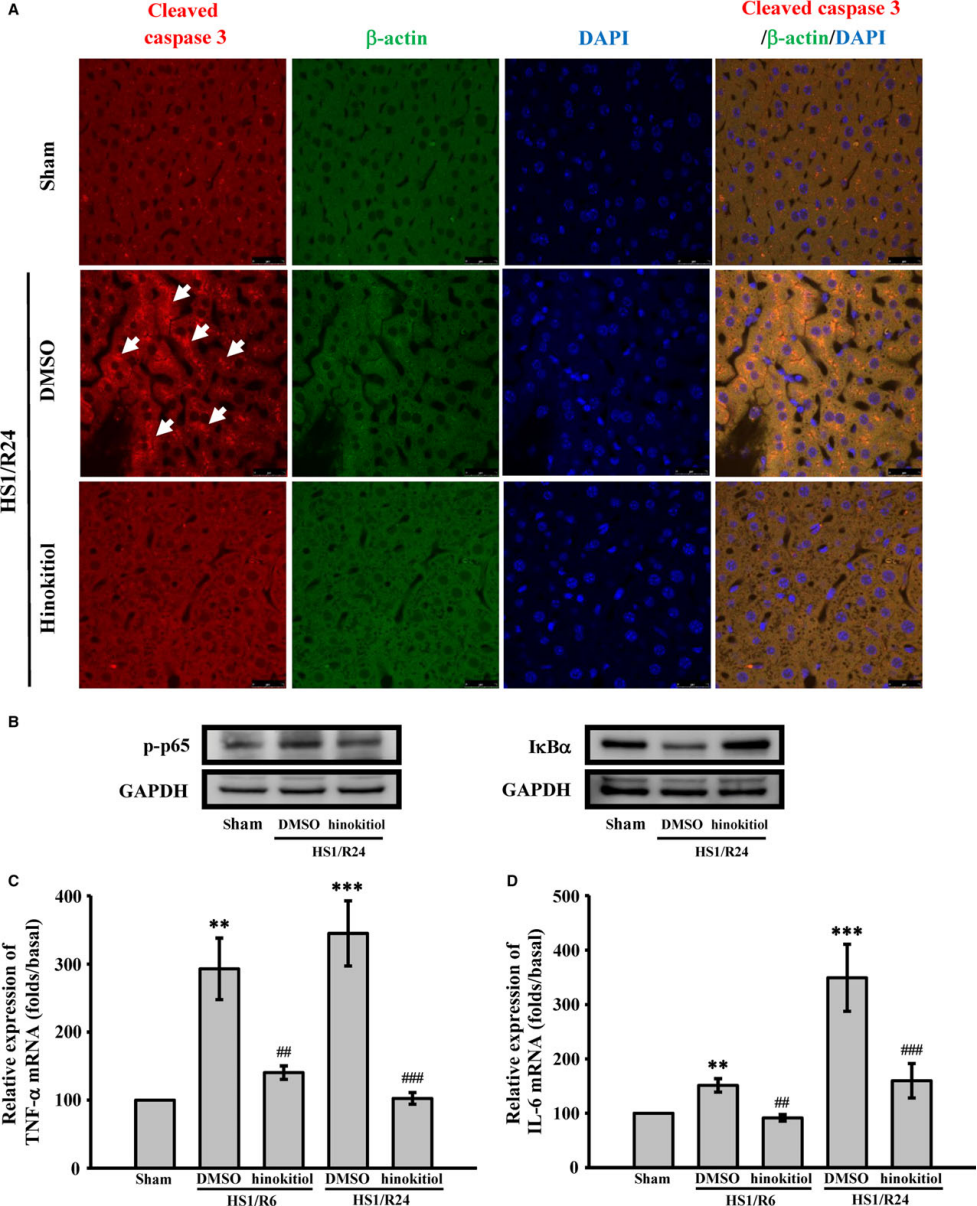
Inhibitory effects of hinokitiol on caspase‐3 activation and inflammatory cytokine production during HS/R‐induced hepatic injury. The mice were subjected to HS1/R6 and HS1/R24. Liver tissues were collected, and the proteins and total RNA were extracted. (A) The cleaved caspase‐3 antibody was used to detect caspase‐3 activation in liver sections by confocal microscopy. Arrows indicate cleaved caspase‐3. Blue depicts the nucleus (DAPI), red depicts cleaved caspase‐3 and green depicts β‐actin. The profiles are representative examples of three similar experiments. The scale bar indicates 40 μm. (B) Immunoblotting was used to measure p65 phosphorylation and IκBα degradation. (C and D) The gene expression of TNFα and IL‐6 were detected using real‐time PCR. Each sample was examined in triplicate, and the amounts of the PCR products produced were normalized to GAPDH, as an internal control. The profile (A and B) is a representative example of three similar experiments. Data in (C and D) are presented as the mean ± SEM (n* *=* *4). **P <* *0.01 and ***P <* *0.001, compared with the sham‐operated group; ##*P *<* *0.01 and ###*P *<* *0.001, compared with DMSO‐treated group subjected to HS/R

In the present study, an in vivo model of HS/R was used to determine whether hinokitiol prevents HS/R‐induced liver damage in mice (Figure 1B). Serum ALT, AST, and LDH levels are crucial indicators of liver dysfunction. Thus, we first observed differences the levels of these three enzymes after HS/R in the absence or presence of hinokitiol. As shown in Figure 2A, B and C, HS for 1 hour followed by resuscitation for 2‐24 hours, resulted in liver injury (indicated by a 2‐ to 4‐fold, a 4‐ to 8‐fold, and a 2‐ to 4‐fold increase in serum ALT, AST and LDH levels, respectively, compared with sham‐operated group). Hinokitiol (1.7 mg/kg) pretreatment revealed a marked decrease in the serum ALT, AST, and LDH levels after HS1/R6 and HS1/R24. Moreover, histological analysis revealed that after HS1/R6 and HS1/R24‐induced liver damage, including hepatocyte ballooning degeneration and loss of sinusoidal architecture and cleaved caspase‐3 expression, which was evidenced by H&E staining and immunohistochemical staining of liver tissues, was obviously reversed by hinokitiol (Figure 2D, E). Moreover, the immunostaining images revealed a marked increase of cleaved caspase‐3 (red; arrows) during HS1/R24‐induced liver damage, which was reversed by hinokitiol (Figure 3A). These findings indicated the protective role of hinokitiol against HS/R‐induced liver injury in vivo.

### 3.2 Hinokitiol attenuates HS/R‐induced hepatic apoptosis in mice

Because cellular apoptosis is a major mechanisms leading to hepatocellular damage during the process of ischaemia/reperfusion,^21^, ^22^ we examined whether hinokitiol prevents hepatic apoptosis after HS/R. As shown in Figure 2F, the levels of several apoptosis‐associated proteins, including cleaved caspase‐3, PARP, Bax and Bcl‐2, were estimated in the liver tissues of mice subjected to HS/R. The data revealed that HS/R24 induced caspase‐3 activation, PARP cleavage, Bax upregulation, and Bcl‐2 downregulation, which were reversed by hinokitiol (1.7 mg/kg). Hinokitiol also reversed the HS/R6‐ and HS/R24‐induced the increase of Bax mRNA (Figure 2G) and decrease of Bcl‐2 mRNA levels (Figure 2H). These findings suggest that hinokitiol prevents HS/R‐induced hepatocellular death partly through apoptosis inhibition.

### 3.3 Hinokitiol prevents HS/R‐induced inflammatory responses in mice

Complication of major trauma by HS/R causes global ischaemia‐reperfusion injury, which results in systemic and end‐organ inflammation and eventually in organ failure. NF‐κB activation induces the expression of pro‐inflammatory mediators, such as TNF‐α and IL‐6, which are involved in the pathogenesis of liver damage after HS/R.^23^, ^24^, ^25^ Thus, NF‐κB activation and the mRNA expression of the inflammatory cytokines TNF‐α and IL‐6 were examined in the liver tissues of mice subjected to HS/R. according to western blotting analysis shown in Figure 3B, HS/R24 markedly increased the phosphorylation of p65 and reduced the degradation of IκBα. Hinokitiol considerably reversed this effect. Moreover, Figure 3C, D revealed that the levels of TNF‐α and IL‐6 mRNA in the groups subjected to HS/R6 and HS/R24 were significantly higher than those in the sham‐operated group; these levels were evidently reduced by hinokitiol. These findings indicate that hinokitiol attenuates HS/R‐induced liver injury, in part, by reducing inflammation.

### 3.4 Hinokitiol attenuates hypoxia/reoxygenation (H/R)‐induced injury in the primary hepatocytes through the reduction of apoptosis

**Figure 4:**
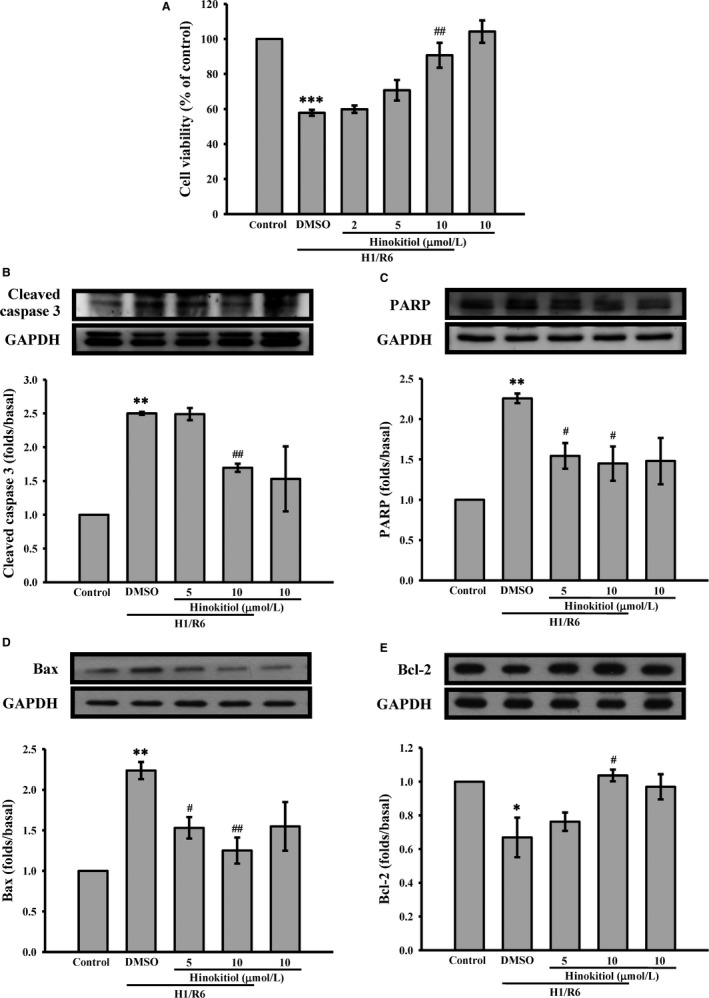
Protective effects of hinokitiol on hypoxia/reoxygenation (H/R)‐induced injury of primary hepatocytes. A, Primary hepatocytes isolated from liver tissue were treated with hinokitiol (2, 5 and 10 μmol/L), and then subjected to H1/R6, and cell viability was then determined by MTT assay. (B‐E) The cells were collected, and subcellular extracts were analysed through immunoblotting. Specific antibodies were used to detect (B) cleaved caspase‐3, (C) PARP, (D) Bax and (E) Bcl‐2. Data are presented as the means ± SEM (n* *=* *4). *P <* *0.05, **P <* *0.01 and ***P <* *0.001, compared with the control group; #*P *<* *0.05 and ##*P *<* *0.01, compared with DMSO‐treated group subjected to H1/R6

**Figure 5:**
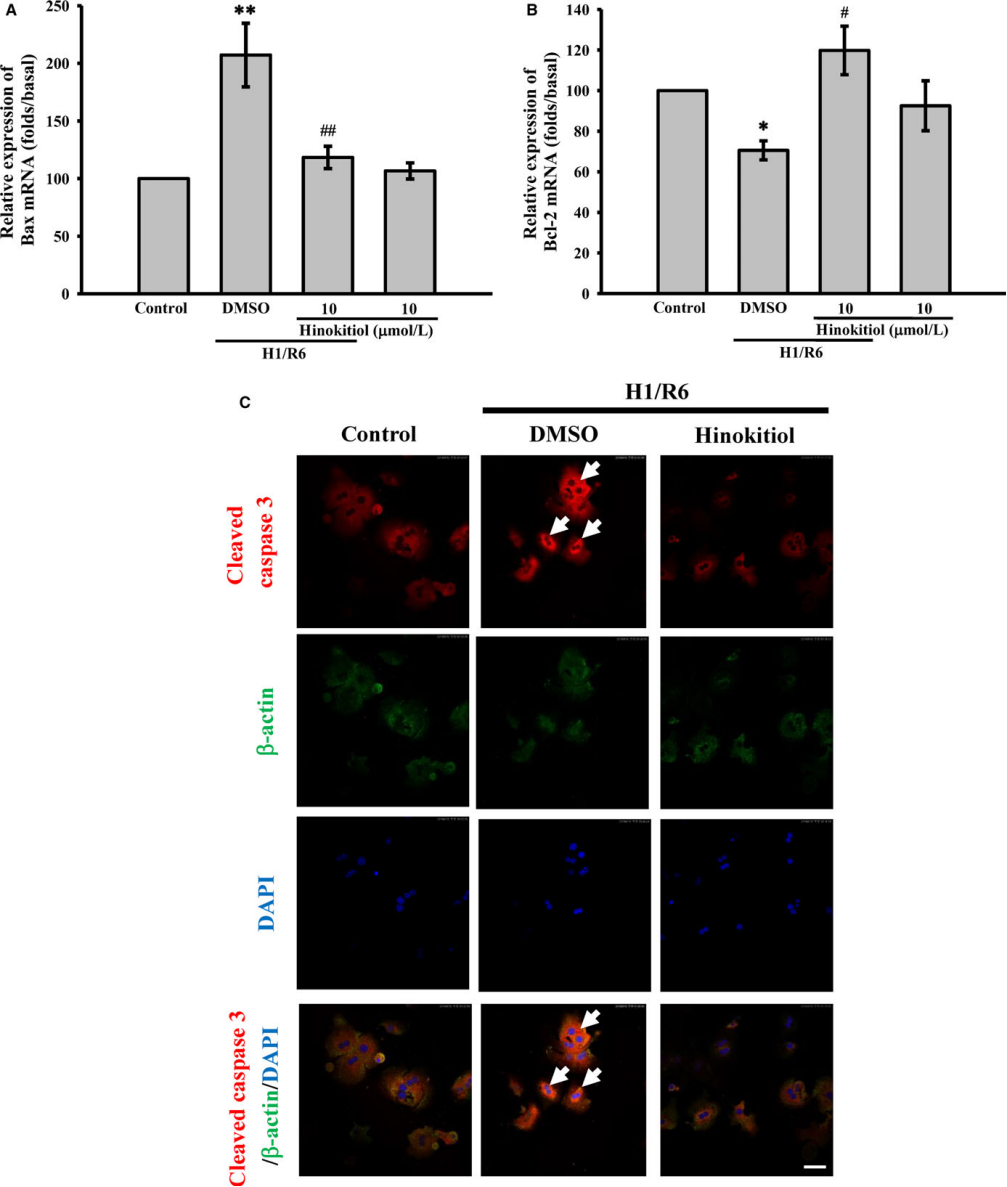
Effects of hinokitiol on H1/R6‐induce apoptosis of primary hepatocytes. Primary hepatocytes were incubated with DMSO or hinokitiol (5 or 10 μmol/L) prior to H1/R6. The total RNA was extracted and converted to cDNA. Specific primers were used to detect the gene expression of (A) Bax and (B) Bcl‐2. Each sample was examined in triplicate, and the amounts of the PCR products produced were normalized to GAPDH, as an internal control. The data are presented as the means ± SEM (n* *=* *4). *P <* *0.05 and **P <* *0.01, compared with the control group; #*P *<* *0.05 and ##*P *<* *0.01, compared with DMSO‐treated group subjected to H1/R6. C, Confocal images obtained in three separate experiments demonstrating caspase‐3 activation (arrows) in the primary hepatocytes. Blue depicts the nucleus (DAPI), red depicts cleaved caspase‐3 and green depicts β‐actin. The white bar indicates 40 μm

In the present study, primary hepatocytes isolation from mice were used to determine the protective effect of hinokitiol on the hepatocytes subjected to the H/R, which has been reported to damage hepatocytes through apoptosis induction. As shown in Figure 4A, the MTT assay revealed that after hypoxia for 1 h and reoxygenation for 6 hours (H1/R6), the cell viability was considerably lower in the control than in the hinokitiol alone group. Pretreatment with hinokitiol (10 μmol/L) significantly inhibited H1/R6‐induced death of hepatocytes. In addition, hinokitiol attenuated caspase‐3 activation (Figure 4B), PARP cleavage (Figure 4C), and Bax protein expression (Figure 4D), and it increased Bcl‐2 protein expression (Figure 4E). Hinokitiol also reduced the level of Bax mRNA (Figure 5A) and enhanced the level of Bcl‐2 mRNA (Figure 5B). Moreover, the immunostaining images revealed marked caspase‐3 activation (red; arrows) during H1/R6 injury, which was reversed by hinokitiol (Figure 5C). These findings suggest that hinokitiol can protect cells from H/R‐induced injury by inhibiting apoptosis.

### 3.5 Hinokitiol suppresses H/R‐induced inflammation by preventing of NF‐κB activation in primary hepatocytes

**Figure 6:**
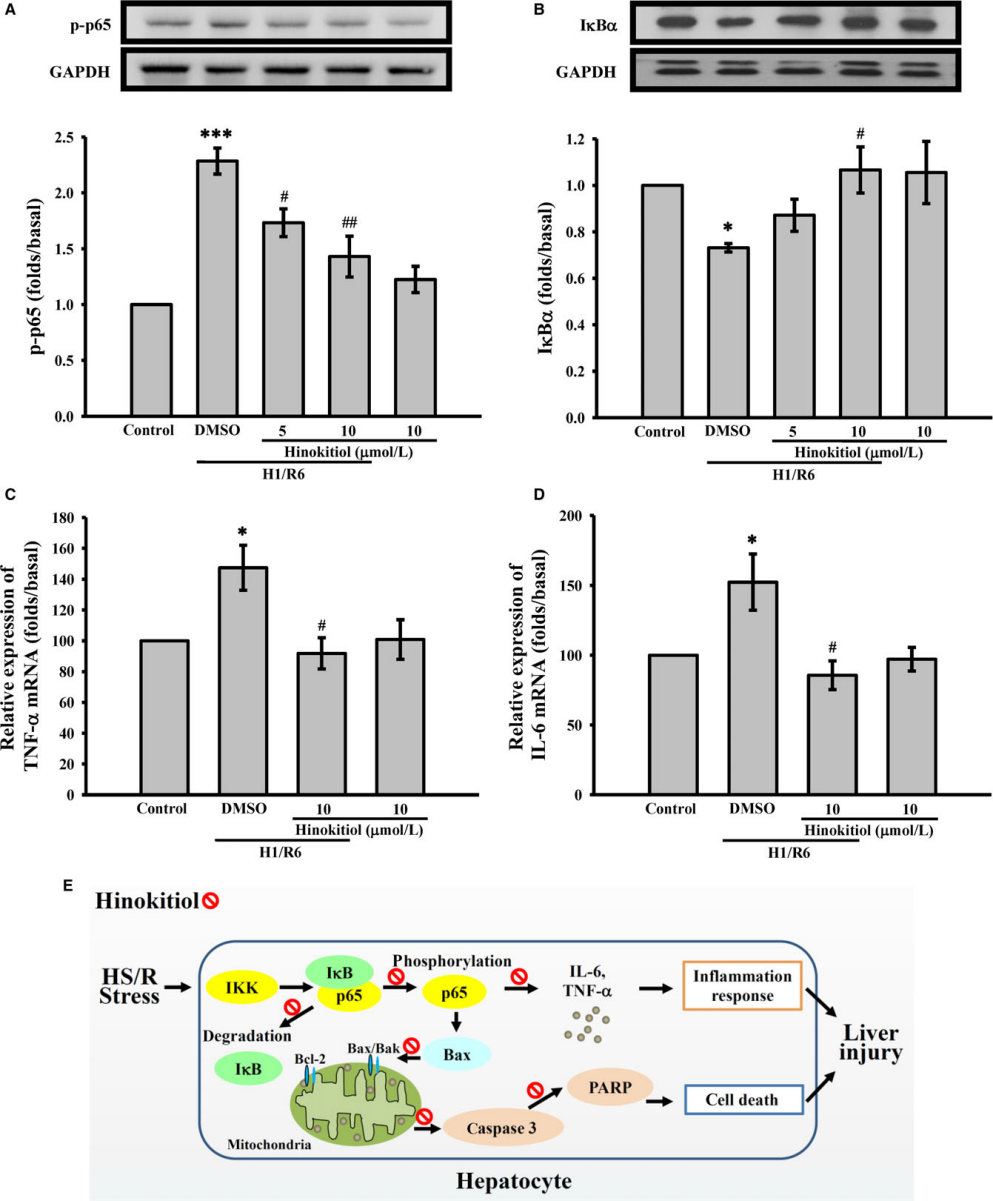
Effects of hinokitiol on H1/R6‐induced inflammation in primary hepatocytes. Primary hepatocytes were incubated with DMSO or hinokitiol (5 or 10 μmol/L) prior to H1/R6. Cells were collected, and subcellular extracts were analysed through immunoblotting. Specific antibodies were used to dectect (A) p65, and (B) IκBα expression. The mRNA levels of (C) TNF‐α and (D) IL‐6 were determined through real‐time PCR. Each sample was examined in triplicate, and the amounts of the PCR products produced were normalized to GAPDH, as an internal control. Data are presented as the means ± SEM (n* *=* *4). *P <* *0.05 and ***P <* *0.001, compared with the control group; #*P *<* *0.05 and ##*P *<* *0.01, compared with DMSO‐treated group subjected to H1/R6. E, Schematic illustration of the hepatoprotective effects of hinokitiol against HS/R injury

H/R‐mediated inflammatory responses are involved in the activation of NF‐κB.^21^ Thus, we further determined the effect of hinokitiol on NF‐κB in the primary hepatocytes. As shown in Figure 6A, B, H1/R6 increased the phosphorylation of p65 and attenuated the degradation of IκBα, which were reversed by hinokitiol in a concentration‐dependent manner. In addition, the expression of NF‐κB downstream target genes, TNF‐α and IL‐6, was determined. The data revealed that hinokitiol significantly reduced the mRNA levels of TNF‐α (Figure 6C) and IL‐6 (Figure 6D) in the hepatocytes subjected to H1/R6. These findings indicate that hinokitiol prevents inflammatory responses through the inhibition of NF‐κB activation.

## 4 DISCUSSION

In recent years, the number of patients at risk of hepatic HS/R injury has been increasing. Research into strategies for the prevention and treatment of hepatic HS/R is urgently required. However, the effect of hinokitiol on hepatic HS/R has not been investigated. The aim of the present study was to evaluate the effect of hinokitiol on HS/R‐induced liver injury and the underlying mechanism. The present study demonstrated for the first time the protective action of hinokitiol against HS/R‐induced liver injury, partly through the suppression of NF‐κB activation, thereby reducing inflammation and apoptosis (Figure 6E). Previously, rat or mouse models of HS/R have been used to investigate trauma haemorrhage‐induced injury. These models reveal that HS/R can cause multi‐organ injury, including kidney, lung and liver injury.^18^, ^26^, ^27^ Among the organs, the liver is currently considered to be most frequently affected by HS.^6^ Similar to their roles in other organs, NF‐κB activation and TNF‐α and IL‐6 production play crucial roles in HS/R‐induced liver injury.^18^, ^26^, ^27^

NFκB, a nuclear transcription factor, plays a critical role in mediating inflammatory and immune responses through its involvement in the production of two pro‐inflammatory cytokines.^23^, ^25^ Notably, NF‐κB signalling related factors, such as IκBα and p65, has been shown to be involved in inflammatory responses caused by ischaemia/reperfusion injury.^28^, ^29^ Moreover, HR‐induced hepatic injury has been reported to consist of two main stages.^6^ The primary injury occurs in the early phase of shock or ischaemia, whereas the secondary injury is attributed to the reperfusion phase. These two phases lead to cytokine production, such as TNF‐α and IL‐6, and neutrophil infiltration, which cause necrosis and apoptosis of hepatocytes.^6^ In our present study, we mainly focused on whether hinokitiol protects against liver injury after HS/R by attenuating inflammatory responses and cellular apoptosis. In this study, an in vivo mouse model of HS/R was used, and hinokitiol was intravenously administered during fluid resuscitation. Indicators of hepatocyte injury, such as serum ALT, and AST levels, play a crucial role in the pathophysiology of hepatic HS/R.^30^ ALT is a specific marker for hepatic parenchymal injury and the AST level is a nonspecific marker for hepatic injury. Our results revealed that hinokitiol ameliorates liver damage, as evidenced by reduced serum levels of ALT and AST and the histological analysis. Moreover, hinokitiol attenuated NF‐κB activation and the expression of pro‐inflammatory cytokines TNF‐α and IL‐6. Simultaneously, hinokitiol also inhibited cellular apoptosis by inhibiting caspase‐3 activation, downregulating Bax, and upregulating Bcl‐2. These findings indicate that hinokitiol can attenuate inflammatory responses and apoptosis, thereby attenuating liver injury after HS/R.

In addition, our in vitro data revealed that hinokitiol protects isolated primary hepatocytes against an H/R injury. Similar to the in vivo data, hinokitiol also suppressed the activation of NF‐κB and of caspase‐3, enhanced Bcl‐2 expression, and attenuated Bax expression. H/R has been reported to induce reactive oxygen species (ROS) production, which can further cause cell apoptosis and damage.^31^ These observations indicate that hinokitiol protects hepatocytes, at least partially, through the suppression of apoptosis due to the generation of ROS during an H/R injury. In addition to anti‐inflammatory activity, our previous studies have demonstrated the potent antiplatelet activity of hinokitiol.^14^, ^15^ Platelet activation induced by trauma and coagulation has been suggested to release pro‐inflammatory mediators that initiate immune response and increase leucocyte‐platelet aggregation, thereby causing endothelial cell damage and subsequently promoting SIRS. In addition, platelet are also a major sources of microvesicles and exosomes, which contain cytokines, metabolites, and lipids that also propagate SIRS.^1^ These observations indicate that the anti‐platelet activity of hinokitiol may also contribute to its hepatoprotective effect following HS/R. In addition, the systemic responses to major trauma are associated with a reduced ability to fight infection, leading to sepsis, and further activation of the destructive inflammatory response; a combination of these responses may cause multi‐organ failure.^1^ Our previous study also indicated that hinokitiol can regulate immune response.^13^ This may offer an additional benefit in trauma‐associated increased susceptibility to infection. Targeting single components or limited pathways has not achieved any reproducible clinical benefits thus far, implying that a multipronged approach may be more beneficial in the clinical setting of trauma.^3^ Therefore, the multiple biological activities of hinokitiol may make it more effective than other drugs for preventing liver injury following HS/R. In conclusion, our data demonstrated that hinokitiol effectively prevented liver injury following HS/R, at least partially, through suppression of inflammation and apoptosis. Our findings also indicate that hinokitiol may serve as a potential therapeutic agent in the clinical setting of trauma.

## CONFLICT OF INTEREST

The authors have no conflicts of interest to declare.

## AUTHOR CONTRIBUTIONS

Wan‐Jung Lu designed and performed experiments and wrote the manuscript. Kuan‐Hung Lin performed the isolation and purification of hepatocytes. Mei‐Fang Tseng and Kuo‐Ching Yuan helped with the animal experiments. Hung‐Chang Huang contributed in the analysis and interpretation of data. Joen‐Rong Sheu and Ray‐Jade Chen supervised the study and revised the manuscript.
